# Adherence to Multidisciplinary Tumor Board Recommendations and Its Association with Survival: A Retrospective Observational Study of Colorectal Cancer Patients

**DOI:** 10.1007/s12029-025-01246-4

**Published:** 2025-05-30

**Authors:** Esin Aysel Kandemir, Julia Roeper, Heiner Zimmermann, Lena Ansmann, Petra Hülper, Maximilian Bockhorn, Claus-Henning Köhne, Frank Griesinger

**Affiliations:** 1 School of Medicine and Health Sciences, University Department Internal Medicine-Oncology, Carl von Ossietzky Universität Oldenburg, Oldenburg, Germany; 2 School of Medicine and Health Sciences, Department of Health Services Research, Carl von Ossietzky Universität Oldenburg, Oldenburg, Germany; 3 Chair of Medical Sociology, Institute of Medical Sociology, Health Services Research and Rehabilitation Science (IMVR), Faculty of Medicine, University of Cologne, Cologne, Germany; 4 Northwest German Tumor Center, Klinikum Oldenburg, Germany; 5 School of Medicine and Health Sciences, University Department for General and Visceral Surgery, Carl von Ossietzky Universität Oldenburg, Oldenburg, Germany; 6 School of Medicine and Health Sciences, University Department for Internal Medicine-Hematology and Oncology, Carl von Ossietzky Universität Oldenburg, Oldenburg, Germany

**Keywords:** Colorectal cancer, Multidisciplinary tumor boards, Survival, Adherence

## Abstract

**Purpose:**

Multidisciplinary tumor boards (MTBs) intend to increase the quality of cancer care. Research on the association of adherence to MTB recommendations with survival is limited. This study aims to determine the impact of adherence to MTB recommendations on survival in colorectal cancer patients.

**Methods:**

This is a retrospective, observational study including patients diagnosed between 01.01.2014 and 31.12.2018. Electronic health records were reviewed to determine the adherence. Study endpoints were adherence rate, disease-free survival (DFS), and overall survival (OS). Follow-up was performed until 12.12.2023.

**Results:**

There was a significant difference in DFS (median DFS: 79 months [95% CI, 73-89] vs 22 months [95% CI, 17-87]) and OS (median OS: 78 months [95% CI, 75-86] vs 65 months [95% CI, 28-NR]) between the adherent group (n=406) versus the non-adherent group (n=52) (log-rank test, p<0.05). Performance status, stage and non-adherence were independent predictors of survival in the multivariate analysis (p<0.05 for all). The most common reason for non-adherence was patient preference (n=23).

**Conclusion:**

While MTBs have become an indispensable part of clinical practice, adherence to MTB recommendations was crucial to achieve survival benefit in this study. Patient preference should be prospectively analyzed from a patient and caregiver perspective in future studies.

## Introduction

Multidisciplinary care of cancer patients is crucial with regular multidisciplinary tumor boards (MTBs), providing an opportunity for joint discussion of patient cases. Colorectal cancer is the third most common cancer worldwide [35] and new cases are estimated to increase from 1.9 million in 2020 to 3.2 million by 2040 [18]. Thus, achieving high-quality health care for these patients is becoming even more important.

Studies comparing outcomes before and after introduction of MTBs or patients with and without MTB discussion in colorectal cancer have demonstrated several benefits: increased survival time [12, 16, 20, 22, 45], increased uptake of neoadjuvant [3, 12, 14, 22] and adjuvant treatment [16, 23], increased access to multimodal care [14], improvement in preoperative staging [3, 40], increase in resection of metastatic liver lesions [12] and in the incidence of R0 resections [22], more complete TNM staging due to increased uptake of magnetic resonance imaging [36], increase in the completeness of total mesorectal excision [28], and change in clinical management plan [7, 33] including addition of neoadjuvant treatment and change in operative approach. However, there are also inconclusive results, demonstrating a decrease in perioperative mortality but no improvement in overall survival (OS) [43].

Adherence to MTB recommendations has been studied in a variety of retrospective and prospective studies and varied between 64–96.3% [1, 2, 6, 11, 20, 21, 31, 33, 41, 44]. Few studies [20], [13], [5, 9, 27] focused on the survival difference between the adherent and non-adherent groups, only one of them including colorectal cancer patients [20]. In this study [20] there was a significant difference in OS-rate at 5 years between MTB patients and non-MTB patients.

A recent study from Germany [11] found that reasons for non-adherence were mostly related to comorbidities and patient preference. However, the influence of adherence and non-adherence on OS remains unknown. In another study, the adherence rate to recommendations from gastroenterological MTBs was 66%, however outcome parameters were not investigated between adherent and non-adherent groups [38]. In this study we aimed to determine the adherence rate to MTB recommendations in colorectal cancer patients, reasons for non-adherence and we hypothesized that adherence to MTB recommendations may lead to improved survival for colorectal cancer patients treated at a German cancer center.

## Method

This is a retrospective observational study of newly diagnosed patients with colorectal cancer between 01.01.2014 and 31.12.2018 in the DKG (Deutsche Krebsgesellschaft/German Cancer Society) certified colorectal cancer center “Klinikum Oldenburg”. Criteria for certification is the discussion of all patients in the MTBs. Klinikum Oldenburg is a tertiary care center affiliated with Carl von Ossietzky University of Oldenburg as a teaching hospital. The hospital is owned by the state and has been certified by the DKG [10] as a colorectal cancer center since 2014. Therefore, all patients with newly diagnosed colorectal cancer were discussed in the MTBs in the study period. It was possible but not necessary to discuss a patient case both in the preoperative and postoperative MTBs. Case volume is estimated as 80 newly diagnosed colorectal cancer cases per year and most of these cases are treated surgically. The study period was determined considering that MTBs were widely implemented in the participating hospital since 2014. Therefore, we aimed to study a period from 2014 until a time that should allow for sufficient follow-up for all patients, also assuring a large sample size. No sample size calculation was performed due to the exploratory nature and the recruitment was maximized as much as possible.

MTBs were organized weekly and resulted in an individualized recommendation for each case. Participation of at least one board certified specialist from surgery, medical oncology, radiation therapy, radiology, and pathology department was necessary. MTB meetings usually lasted between 1–2 hours, depending on how many patients were presented at the meeting. Preoperative MTBs aimed at assessing the patient in terms of operability, resection of metastases if applicable and determining neoadjuvant treatment. Postoperative MTBs focused on treatment planning after surgery/resection. Preoperative and postoperative MTBs were held at the same MTB session and was classified as either “preoperative” or “postoperative” considering each patient’s surgical history for initially diagnosed colorectal cancer. Patients without follow-up data (no information available in the medical records after MTB decision), patients with no specific MTB recommendation (recommendation refers to only diagnostic approaches and no recommendation was made regarding the management of the disease) and patients who died within 30 days of diagnosis, were excluded from the analysis. Pre- and postoperative MTB records, patients’ histories, physicians’ letters, pathology and screening results and follow-up data were retrospectively reviewed. MTB discussion had been documented by the hospital personnel using a template. In rare cases, MTB discussion was written in the medical notes of the patients in a free format. Staging was implemented in accordance with the American Joint Committee on Cancer (AJCC) TNM classification system 7 th edition, which was valid at that time. To reduce bias, adherence and non-adherence were determined in a systematic way by the first author and for challenging cases the senior author was consulted for a decision. Adherence to MTB recommendations was defined as follows:

- Adherence: All recommendations were implemented in clinical practice.
- Non-adherence: At least one recommendation was not implemented in clinical practice.

If the recommendation was not precise and suggested consideration of therapeutic modalities depending on comorbidities and general health status of the patients by the treating physician, either administration of the recommended treatment or a decision for not administering the recommended treatment was accepted as adherent. The reasons for non-adherence were captured if they were documented in the patients’ electronic health records and classified into four categories:

1. Patient preference: The patient did not want to receive the recommended treatment or denied communication attempts (phone call, communication via relatives, etc).
2. Physician’s choice: The treating physician preferred another treatment without a change in clinical situation after MTB.
3. Altered treatment indication: Treatment indication altered after MTB due to a change in the clinical situation.
4. Absolute contraindication
5. Relative contraindication
6. Restaging
7. Rapid disease progression
8. Unknown: No reason was documented in the electronic health records.

Deviation from recommended treatment regarding chemotherapy cycles, administered doses and switch from 5-fluorouracil to capecitabine or vice versa were considered adherent. Cases in which the MTB recommended observation and the patient was followed-up without treatment were considered adherent. Pius Hospital is a tertiary cancer center which provides therapy in the inpatients and outpatients setting. External practices are practices in the towns with oncologists administering therapy in an ambulatory setting and provide independent oncological care.

The primary endpoints were adherence rate and disease-free survival (DFS). Adherence rate is the proportion of the number of adherent cases to the number of all cases. DFS is the time from first diagnosis until disease recurrence or death in stage I-III patients, whichever occurred first. Cases without disease recurrence, were censored at the time point of last known follow-up by medical records. The secondary endpoint was OS. OS was defined as the time from first diagnosis until death due to any cause. Cases still living at last follow-up were censored at that time point. Kaplan-Meier survival analysis was used for the estimation of the survival endpoints. Median follow-up time was calculated using the reverse Kaplan-Meier method. Based on clinical judgement; age, stage, ECOG performance status (PS) were considered as potential confounders, and included in the multivariate cox proportional hazards (CPH) model together with family status and healthcare organization. Age (≤60 years, 61–75 years, ≥76–94 years) and ECOG PS (0, 1, other) were categorized in the CPH models considering clinical meaning and comparable number of patients in the categories. Proportionality of hazards was checked by Schoenfeld residuals test for each variable in the model and variables not meeting the proportional hazards assumption were stratified in the no-interaction CPH model. Chi-square test was used to test the difference in categorical variables (sex, family status, health insurance, ECOG PS, tumor site and tumor localization, stage, grade, microsatellite instability status, healthcare organization) between groups. Wilcoxon-Mann-Whitney test was used to test the difference in numerical variables with non-normal distribution (age, Charlson comorbidity index) between groups. The significance level was set at 5%. Patients were followed-up until 12.12.2023. Any missing data was recorded as unknown and was included in the analysis. R version 4.3.3 [25] and RStudio version 2023.12.1.402 [24] were used for the statistical analysis. Statistical packages used in the analysis include survival [37], survminer [8], and gmodels [42] packages. Ethics approval was obtained from the Ethical Commission of Carl von Ossietzky University of Oldenburg (2018-100). Patients were primary cases of the cancer center (Klinikum Oldenburg) and consented to the analyses within the tumor center. Patient data were pseudonymized before analysis and the study was performed in accordance with the Declaration of Helsinki. This article is written in compliance with the STROBE statement [39].

## Results

Six hundred forty-nine cases were identified. After removing duplicated cases with the same patient identification number (n=14), 635 cases remained. Of these, 177 were excluded due to lack of discussion at the MTB or lack of MTB documentation (n=121), death of patient within 30 days of diagnosis (n=26), lack of healthcare data (n=25), and other reasons (n=5). This resulted in the inclusion of 458 colorectal cancer patients in the study. Median follow-up time (95% confidence interval [CI]) was 70 months (95% CI, 65–76) and 68 months (95% CI, 62–72) for DFS and OS; respectively. Descriptive statistics including patient characteristics are presented in Table 1. Most patients received treatment adherent to the MTB recommendation (88.6%). The most common reason for non-adherence was patient preference (23 out of 52 non-adherent patients, 44.2%). Surgery was the predominant approach in the preoperative MTBs (48.2%, n=221) which was followed by neoadjuvant treatment (11.3%). All neoadjuvant treatment recommendations were implemented (n=52) and one patient received neoadjuvant treatment without MTB recommendation. Frequent recommendations in the postoperative MTBs were follow-up (46.5%, n=213) and adjuvant treatment (29.7%). Adjuvant treatment was recommended in 136 patients, of which 100 patients were adherent to the recommended adjuvant treatment and 30 patients were non-adherent. The remaining 6 patients had a recommendation that was not precise and suggested consideration of adjuvant treatment depending on comorbidities and general health status, which were considered as adherent. Further details of MTB recommendations are summarized in Table 2.

**Table 1:** Descriptive statistics of the study population

		Adherence (n=406)	Non-adherence (n=52)	Total (n=458)	p-value
Age (years, mean ± SD)		66.03 ± 12.4	66 ± 12.4	66 ± 12.4	p=0.90^a^ (W-statistic: 10448)
Sex	Female	166 (40.9%)	20 (38.5%)	186 (40.6%)	p=0.85^b, c^ (df: 1, χ^2^-statistic: 0.03)
	Male	240 (59.1%)	32 (61.5%)	272 (59.4%)	
Family status	Married	196 (48.3%)	18 (34.6%)	214 (46.7%)	p=0.10^b^ (df: 2, χ^2^-statistic: 4.5)
	Single	86 (21.2%)	11 (21.1%)	97 (21.2%)	
	Unknown	124 (30.5%)	23 (44.2%)	147 (32.1%)	
Health insurance	State	328 (80.8%)	44 (84.6%)	372 (81.2%)	p=0.63^b,c^ (df: 1, χ^2^-statistic: 0.2)
	Private	78 (19.2%)	8 (15.4%)	86 (18.8%)	
ECOG PS	0	231 (56.9%)	26 (50%)	257 (56.1%)	p=0.64^b^ (df: 2, χ^2^-statistic: 0.9)
	1	142 (35%)	21 (40.4%)	163 (35.6%)	
	Other (2–3)	33 (8.1%)	5 (9.6%)	38 (8.3%)	
CCI (median [min, max])		3 (0, 11)	3 (0, 9)	3 (0–11)	p=0.93^a^ (W-statistic: 10637)
Histology	Adenocarcinoma	402 (99%)	52 (100%)	454 (99.1%)	-
	Other	4 (0.1%)	-	4 (0.9%)	
Tumor site	Colon	243 (59.9%)	25 (48.1%)	268 (58.5%)	p=0.14^b,c^ (df: 1, χ^2^-statistic: 2.2)
	Rectum	163 (40.1%)	27 (51.9%)	190 (41.5%)	
Tumor localization (Rectum)	Upper part	42 (10.3%)	4 (7.7%)	-	p=0.52^b,d^ (df: 2, χ^2^-statistic: 1.3)
	Middle part	100 (24.6%)	18 (34.6%)	-	
	Lower part	21 (5.2%)	4 (7.7%)	-	
	Unknown	-	1 (1.9%)	-	
Tumor localization (Colon)	Right	142 (35%)	14 (26.9%)	-	p=1^b,c,d^ (df: 1, χ^2^-statistic<0.001)
	Left	93 (22.9%)	9 (17.3%)	-	
	Unknown	8 (2%)	2 (3.8%)	-	
Stage	I	133 (32.8%)	3 (5.8%)	136 (29.7%)	p<0.001^b^ (df: 3, χ^2^-statistic: 17.6)
	II	94 (23.1%)	15 (28.8%)	109 (23.8%)	
	III	95 (23.4%)	21 (40.4%)	116 (25.3%)	
	IV	84 (20.7%)	13 (25.9%)	97 (21.2%)	
Grade	1	9 (2.2%)	2 (3.8%)	11 (2.4%)	p=0.74^b,d^ (df: 2, χ^2^-statistic: 0.6)
	2	322 (79.3%)	39 (75%)	361 (78.8%)	
	3	73 (18%)	9 (17.3%)	82 (17.9%)	
	Unknown	2 (0.5%)	2 (3.8%)	4 (0.9%)	
MSI Status	MSI-High	21 (5.2%)	4 (7.7%)	25 (5.4%)	p=0.67^b^ (df: 2, χ^2^-statistic: 0.8)
	MSI-Low	92 (22.7%)	13 (25%)	105 (23%)	
	Unknown	293 (72.2%)	35 (67.3%)	328 (72%)	
Healthcare organization	Pius Hospital	6 (1.5%)	4 (7.7%)	10 (2.2%)	p=0.009^b^ (df: 2, χ^2^-statistic: 9.5)
	Klinikum Oldenburg	122 (30%)	11 (21.1%)	133 (29%)	
	External practice	278 (68.5%)	37 (71.1%)	315 (68.8%)	
Status	Recurrence rate (n=361 patients)	134/322 (41.6%)	26/39 (66.7%)	160/361 (44.3%)	-
	Death rate	158 (38.9%)	26 (50%)	184 (40.2%)	-

CCI: Charlson comorbidity index, df: degree of freedom, ECOG PS: Eastern Cooperative Oncology Group Performance Status, MSI: Microsatellite instability, SD: Standard deviation. ^a^Wilcoxon-Mann-Whitney test, ^b^Chi-square test. ^c^Chi-square test with Yates’ continuity correction. ^d^Observations with unknown values are excluded before performing statistical test.

**Table 2:** Summary of adherence status and MTB recommendations

		N=458 (100%)
Adherence status	Adherence	406 (88.6%)
	Non-adherence	52 (11.4%)
Preoperative MTB recommendation	Yes	310 (67.7%)
	- Surgery	221 (48.2%)
	- Neoadjuvant treatment	52 (11.3%)
	- Treatment with palliative intent (ChT, RT or CRT)	28 (6.1%)
	- BSC	1 (0.2%)
	- Other	8 (1.7%)
	No	148 (32.3%)
Postoperative MTB recommendation	Yes	432 (94.3%)
	- Follow-up	213 (46.5%)
	- Adjuvant treatment	136 (29.7%)
	- Treatment with palliative intent (ChT, RT or CRT)	60 (13.1%)
	- BSC	10 (2.2%)
	- Other	13 (2.8%)
	No	26 (5.7%)
Systemic treatment	Neoadjuvant	53 (11.6%)
	- Capecitabine	32 (7%)
	- 5-fluorouracil	14 (3.1%)
	- Other	7 (1.5%)
	Adjuvant	100 (21.8%)
	- FOLFOX	37 (8.1%)
	- Capecitabine	41 (8.9%)
	- 5-fluorouracil	14 (3.1%)
	- Other	8 (1.7%)
	Palliative	67 (14.6%)
	- FOLFOX	10 (2.2%)
	- Capecitabine	1 (0.2%)
	- FOLFIRI	17 (3.7%)
	- FOLFOXIRI	7 (1.5%)
	- ChT + Bevacizumab	11 (2.4%)
	- ChT + AntiEGFR monoclonal antibody	18 (3.9%)
	- Other	3 (0.6%)
Reasons for non-adherence		(n=52)
Patient preference (n=23, 44.2%)	Refused ChT	16 (30.8%)
	Refused CRT	3 (5.8%)
	Refused any type of medical care	2 (3.8%)
	Refused surgery	1 (1.9%)
	Refused any communication attempt	1 (1.9%)
Alteration in treatment indication (n=7, 13.5%)	Rapid disease progression	3 (5.8%)
	Absolute contraindication	1 (1.9%)
	Relative contraindication	2 (3.8%)
	Re-staging	1 (1.9%)
Physician’s choice (n=2, 3.8%)	-	2 (3.8%)
Unknown (n=20, 38.5%)	-	20 (38.5%)

AntiEGFR: Anti-epidermal growth factor receptor, BSC: Best supportive care, ChT: Chemotherapy, CRT: Chemoradiotherapy, FOLFIRI: 5-fluorouracil, folinic acid, irinotecan, FOLFOX: 5-fluorouracil, folinic acid, oxaliplatin, FOLFOXIRI: 5-fluorouracil, folinic acid, oxaliplatin, irinotecan, RT: Radiotherapy

**Fig. 1:**
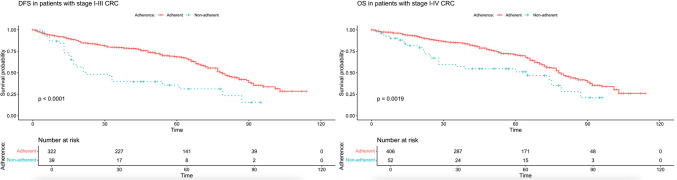
Kaplan-Meier estimates of DFS in patients with stage I-III CRC (n=361) and OS in patients with stage IV CRC (n=458) (survival time in months)

**Fig. 2:**
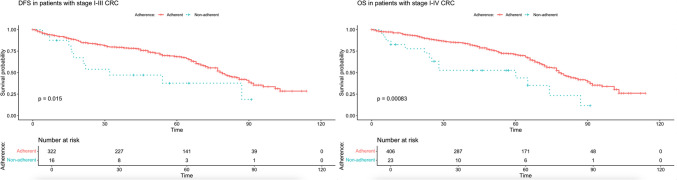
Kaplan-Meier estimates of DFS in patients with stage I-III CRC (n=338) and OS in patients with stage IV CRC (n=429) with the non-adherence group only including cases with patient preference as a reason (survival time in months)

There was a significant difference in DFS (median DFS: 79 months [95% CI, 73–89] vs 22 months [95% CI, 17–87]) and OS (median OS: 78 months [95% CI, 75–86] vs 65 months [95% CI, 28-NR]) between adherent and non-adherent patients (log-rank test, χ^2^ (1) = 19.8, p=0.000009 and χ^2^ (1) = 9.6, p=0.002; respectively). We also performed the survival analysis with the non-adherent group only including cases with patient preference as a reason (n=23) to eliminate the confounding factors from medically justified reasons (altered treatment indication and physician’s choice) and unknown reasons. Survival difference between adherent and non-adherent groups in terms of DFS and OS (median DFS: 79 months [95% CI, 73–89] vs 32 months [95% CI, 17-NR], median OS: 78 months [95% CI, 75–86] vs 60 months [95% CI, 25-NR]) was maintained in this analysis (log-rank test, χ^2^ (1) = 5.9, p=0.02 and χ^2^ (1) = 11.2, p=0.0008; respectively). Kaplan-Meier survival curves for DFS and OS are provided in Figure 1 and Figure 2, respectively.

**Fig. 3:**
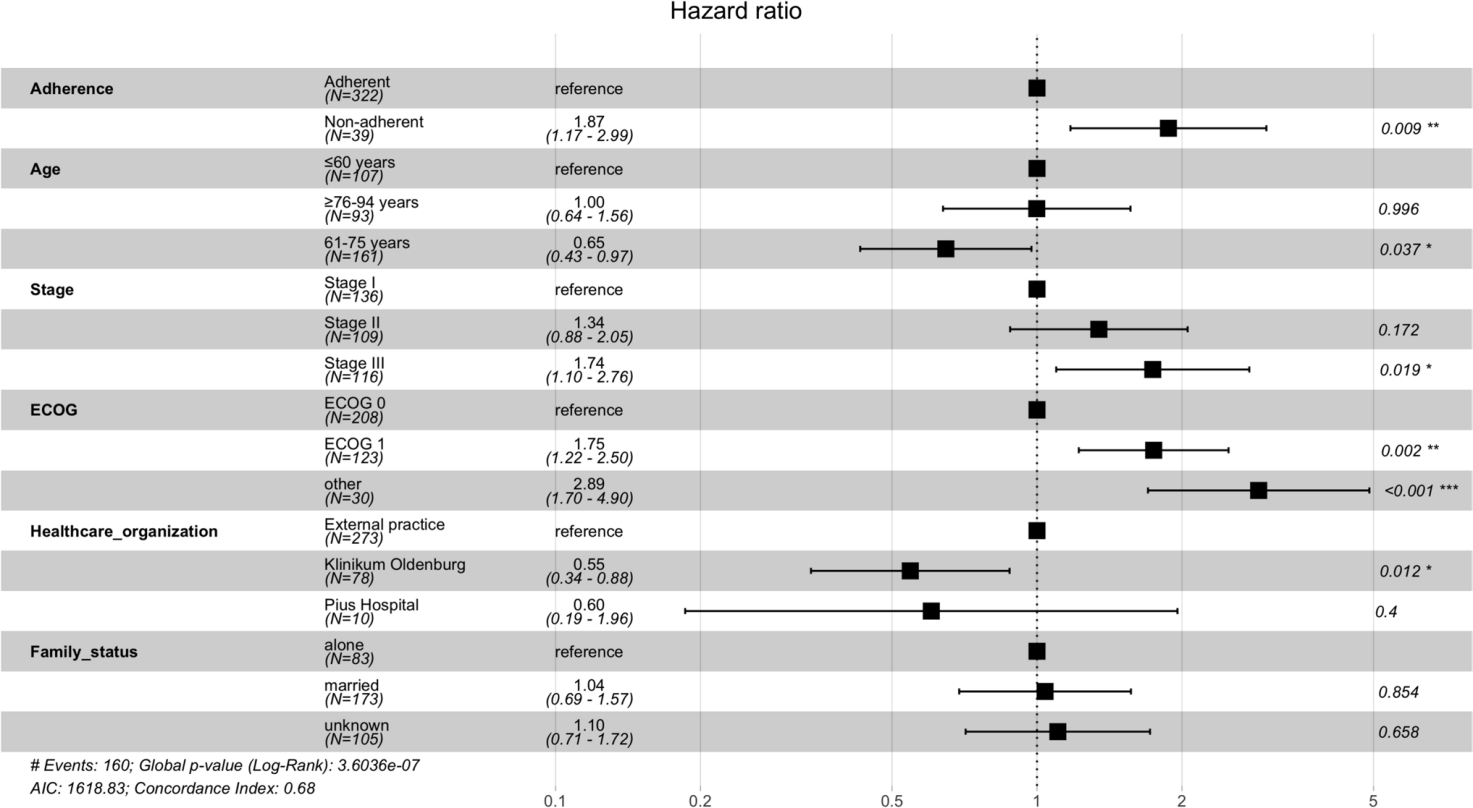
Forest plot of CPH model for DFS in patients with stage I-III CRC (n=361)

**Fig. 4:**
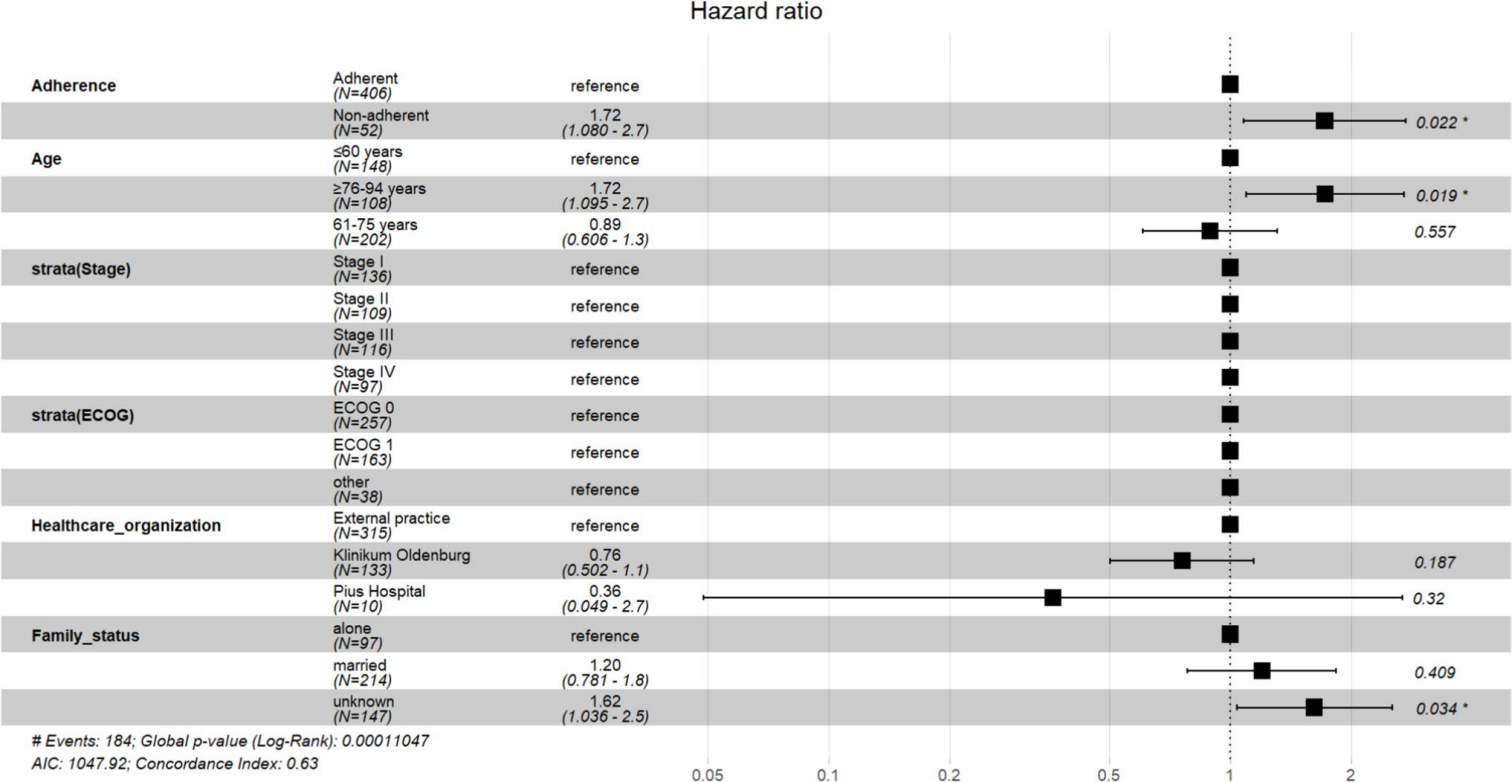
Forest plot of stratified CPH model for OS in all patients (n=458)

The multivariate CPH models for DFS and OS were adjusted for age, stage, ECOG PS, family status, and healthcare organization, to examine whether adherence or non-adherence were independent prognostic factors. Based on the results from Schoenfeld residuals test CPH model for OS was stratified by stage and by ECOG PS. All other variables met the proportional hazards assumption. In the CPH model for DFS independent predictors were as follows: non-adherence, age within 61–75 years, stage III disease, ECOG PS≥1, receiving treatment at Klinikum Oldenburg (Figure 3). In the stratified CPH model for OS independent predictors were non-adherence, age within 76–94 years and unknown family status (Figure 4).

## Discussion and Conclusions

This study demonstrated the association of adherence to MTB recommendations with DFS and OS in colorectal cancer patients, highlighting the importance of adherence to MTB recommendations in cancer care. In our cohort, the majority of MTB recommendations (88.6%) was implemented. This rate is within the range of the results from previous studies [11, 20, 44]. In the study of Krause et al [11], the adherence rate was 69% in colorectal cancer patients (n=283) in Germany. The differences between adherence rates may be explained by institutional factors and methods of adherence assessment. One of the most relevant questions was, whether non-adherence was an independent prognostic factor for survival. This could be demonstrated by a statistically significant difference in DFS and OS in the multivariate analysis, which included prognostically relevant parameters such as age, stage, and ECOG PS.

The strengths of this study are the primary and secondary outcomes, defined as the survival time of the patients and comparing survival time between adherent and non-adherent cases. Thirteen studies considered survival time as an outcome [4, 12, 15–17, 19, 20, 22, 29, 30, 32, 43, 45] in all of which survival comparison was made between MTB patients and non-MTB patients, but not in relation to the adherence. One drawback of the studies investigating the impact of MTBs and comparing outcomes between pre- and post-introduction of MTBs might be the predisposition to a chronological bias because of the advances in cancer care [34]. A similar study to ours [20] included patients who were not discussed in the MTB as well as who were discussed in the MTB but had a non-adherent treatment thereafter, in the non-MTB group. There was a significant difference in OS-rate at 5 years when two groups were compared [Patients who were discussed in the MTB and had an adherent treatment to the MTB recommendation (n=411) vs patients who were not discussed in the MTB (n=73) combined with patients who had a non-adherent treatment after MTB discussion (n=102): 52.2% vs 33.6%,p<0.00001]. This approach, however, prevented a direct comparison between adherent and non-adherent cases. Furthermore, patients in the non-MTB group were older (75.3 ± 1.6 years) compared to the patients in the MTB group (68.6 ± 1.2 years) and there was no statistically significant OS difference if only the patients who survived at least 6 weeks after diagnosis were included in the analysis (OS-rate at 5 years: 63.2% vs 57.7%, p=0.064) [20]. In contrast, all patients in our study were discussed in the MTB and differences in survival were analyzed solely with respect to the adherence status. This approach preserves homogeneity in the overall population and makes our study unique. Furthermore, our study included a relatively large number of patients compared to other studies investigating only 300 patients [16], [22], [15].

The most frequent reason for non-adherence was patient preference (44.2%, n=23 of 52 patients), similar to what was reported in other studies, with a prevalence ranging from 14.8% (4 of 27 patients) [21], 16% (24 of 151 MTB recommendations) [11] to a maximum sum of 28.3% (17 of 60 patients) [41]. Non-adherent cases with unknown reasons were reported in the literature between 20% (12 of 60 patients) [41] and 58% (44 of 76 patients) [20] as in our study. This shows that our findings (38.5%, 20 of 52 patients) are most likely in line with published data from other studies with different healthcare systems and cultural backgrounds. Beyond patient preference and unknown reasons, all other reasons were medically justified including alteration in treatment indication (n=7) and physician’s choice (n=2). Better determination and understanding of patient preferences by the application of quantitative and qualitative research methods could potentially increase the acceptance rate of recommendations and thus improve the outcome of these patients. On the other side, patients should be respected if they refuse the treatment provided that patient preferences rely on well-informed decisions.

There are some limitations to consider. Firstly, this study is a retrospective observational study and therefore potentially prone to selection bias. Cases with recommended follow-up were classified as adherent as long as there was no documentation stating follow-up care was not provided, although they may in fact have not received the required medical check-ups. Secondly, this study only investigates the effect of adherence to MTB recommendations regarding the initial treatment after diagnosis. Decisions regarding treatment in later lines were usually not subject to MTB discussion and were therefore not part of this analysis. Thirdly, there were more patients in the adherence group, which might have influenced the outcome of the study. However, it is impossible to allocate even number of patients to the groups due to the real-life study design and physicians’ and patients’ tendency to follow the recommendations from MTBs, especially in case of malignant diseases. Another issue is the lack of sample size calculation in this study. The findings of this study should be taken as preliminary and can be used to design prospective studies with sample size calculation to compare survival time between adherent and non-adherent patient groups. Not all recommendations from the MTBs necessarily increase survival time, such as palliative radiotherapy in case of painful bone metastases and best supportive care measures if the patient is not fit enough to receive any treatment. The study protocol stipulated that these cases are to be included in the adherence group. Even with their inclusion (n=8 colon and n=3 rectum cancer patients; n=11 in total) in the adherence group, the positive impact of adherence on survival time was still maintained.

Another potential limitation is the exclusion of approximately 23% of the available patient cases due to lack of MTB discussion or lack of MTB documentation (n=121) and lack of healthcare data (n=25), although these data belonged to a certified cancer center. Other studies also reported lack of documentation [36, 38]. For instance, circumferential resection margin was only available in 61% of the pathology reports in one study [36], which highlights that accurate documentation is indispensable for the quality of clinical research.

Future studies prospectively assessing survival time of patients as primary and secondary endpoints are needed to confirm the results of this study. Moreover, studying patient-related outcomes such as quality of life should be considered. Another factor, which was not addressed in our study but can have an effect on health-related outcomes are the characteristics of the organization, team climate, and participation of various disciplines in the MTBs [26],and its impact on survival time of the patients. Furthermore, as new information accumulates in a rapid way in medical literature and treatment options increase, MTBs are becoming more specialized and integrated into clinical practice, focusing on patients with specific health care needs, such as patients with liver metastases, oligometastasis, rare mutations, and young colorectal cancer patients. Future research should focus on these specific types of MTBs investigating adherence rate and survival time as outcome measures.

## Data Availability

The dataset generated and analyzed during the current study is available from corresponding author on reasonable request.
